# Morphological, biochemical and histological effects of aqueous extracts of peanut (*Arachis hypogaea*) on swiss mice in different diets

**DOI:** 10.1590/ACB360905

**Published:** 2021-11-08

**Authors:** Thárcia Kiara Beserra de Oliveira, Josivanda Palmeira Gomes, Paulo Roberto da Silva, Amelia Ruth Nascimento Lima, Ana Janaina Jeanine Martins de Lemos Jordão, Katharina Rodrigues de Lima Porto Ramos, Joelmir Lucena Veiga da Silva, Chirlaine Cristine Gonçalves

**Affiliations:** 1 DVM, PhD. Postgraduate Program of Agricultural Engeneering - Department of Technology and Natural Resources - Universidade Federal de Campina Grande (UFCG) - Campina Grande (PB), Brazil. Assistant Professor. Division of Surgical Skills - Surgical Technique I and II - Faculdade de Medicina de Olinda (FMO) – Olinda (PE), Brazil.; Faculdade de Medicina de Olinda, Olinda, PE, Brazil; 2 PhD. Postgraduate Program of Agricultural Engeneering - Department of Technology and Natural Resources - Universidade Federal de Campina Grande (UFCG) - Campina Grande (PB), Brazil.; 3 Graduate student. Department of Medicine - Centro Universitário Unifacisa – Faculdade de Ciências Médicas de Campina Grande - Campina Grande (PB), Brazil.; 4 MSc student. Department of Natural Sciences and Biotechnology – Universidade Federal de Campina Grande (UFCG) - Campina Grande (PB), Brazil.; 5 PhD. Department of Animal Bioscience - Universidade Federal Rural de Pernambuco (UFRPE) - Recife (PE), Brazil.; 6 Fellow PhD degree. Postgraduate Program of Processing Engeneering - Department of Pharmaceutical Sciences - Universidade Federal de Campina Grande (UFCG) - Campina Grande (PB), Brazil.; 7 PhD. Department of Natural and Synthetic Bioactive Products - Universidade Federal da Paraíba (UFPB) - João Pessoa (PB), Brazil.; 8 PhD. Postgraduate Program of Processing Engeneering - Department of Sciences and Technology - Universidade Federal de Campina Grande (UFCG) - Campina Grande (PB), Brazil.

**Keywords:** Arachis hypogaea, Peanuts, Skin, Dyslipidemias, Mice

## Abstract

**Purpose:**

To evaluate the morphological, biochemical, and histological effects of aqueous extracts of peanut (skinless and added to 1% skin) in Swiss mice submitted to a high-fat diet.

**Methods:**

Forty male Swiss mice were divided into four groups (n=10 per group): GI) normocaloric diet; GII) high-fat diet; GIII) high-fat diet + 0.5 mL of peanut extract; GIV) high-fat diet + 0.5 mL of peanut extract + 1% peanut skin. The animals were weighed weekly and euthanized after 12 weeks for histopathological and biochemical analyses. The study was approved by the Animal Use Ethics Committee.

**Results:**

The animals in the GIV group had higher body weight when compared to the other ones. Increase in total cholesterol in GIII, increase in blood glucose in groups GII, GIII and GIV, decrease in serum low-density lipoprotein (LDL) concentration in groups GI and GIV and increase in serum concentration of C-reactive protein in GII were seen. The presence of vacuolar fat deposits was found in animal livers from GII.

**Conclusions:**

The extracts improved the plasma concentrations of animals that received a high-fat diet, including preventing morphological damage to liver tissue. These benefits were enhanced by the association of peanut shells with the extract.

## Introduction

The development of society in recent decades has changed the popular diet, increasing the demand for food rich in functional nutrients, of good quality and low cost. In Brazil, it is estimated that 10 million people suffer from problems related to inadequate nutrition. A balanced diet contributes to the prevention of diseases such as systemic arterial hypertension, hypercholesterolemia and obesity^1^^,^ 2.

The population’s diet, in general, has been poor in essential nutrients. This is due in part to changes in social dynamics, making people prefer fast meals, rich in carbohydrates and lipids. Thus, there is the need for new research on alternative food that benefits the general population^3^.

In this scenario, the by-products of food processing become a great potential with economic interest. The edible parts of the peanut consist of the almond and the protective skin. The skin, which is red-pink in color and has an astringent taste, is normally removed. However, it contains phenolic compounds, dietary fibers and other health-promoting agents^4^.

It is suggested that polyphenols derived from peanut skin confer resistance to Western diet-induced hyperlipidemia in rats. This may have broader implications for harnessing peanut skin, which, being a great source of bioactive phenolic compounds, may become an ingredient in food industry^5^. In addition, peanut skin is a source of vegetable protein, dietary fiber, antioxidant vitamins, minerals (selenium, magnesium, and manganese), and phytochemicals such as resveratrol and other polyphenols^6^^-^ 8.

The aqueous extract of peanut is still poorly studied, but it is known to have a very high-protein content, with a high index of antioxidant components that act in increasing high-density lipoprotein (HDL) levels and decreasing the serum concentration of low-density lipoprotein (LDL)^6^^-^ 8. Thus, there is a viable hypothesis for the physiological interference of peanut by-products in lipid regulation.

Since the aqueous extract of peanuts and peanut skin are new products, there are still lacking studies regarding their use in the prevention and treatment of lipid and glycemic alterations. Thus, the objective of this study was to evaluate the effects of aqueous extracts of peanut (skinless and added to 1% skin) in the metabolism of Swiss mice submitted to a high-fat diet, through the analysis of body weight gain, serum biochemistry, and histopathological analysis of the heart and liver.

## Methods

This is an experimental study, carried out according to the current rules of the Brazilian National Council of Animal Experimentation and the Brazilian College of Animal Experimentation. This was followed by the Brazilian Practice Guideline for the Care and Use of Animals for Scientific and Didactic Purposes and the Animal Research: Reporting of In Vivo Experiments (ARRIVE) guideline. The experiment was carried out after approval by the Ethics Committee on the Use of Animals of the Centro de Ensino Superior e Desenvolvimento, under protocol number 6727052016.

The work was conducted at the Agricultural Products Storage and Processing Laboratory of the Agricultural Engineering Academic Unit of Universidade Federal de Campina Grande, in the *vivarium* of the Unifacisa University Center, Universidade Estadual da Paraíba, and the Medical School of Olinda.

Forty albino male mice of the Swiss strain, weighing between 25 and 30 g, from the colony of creation of the vivarium, were used. The animals were housed in polypropylene cages with a dimension of 430 × 430 × 200 mm, in an environment with the temperature of 23±1°C and a light/dark cycle of 12 h. Animals had free access to water. The experiment started when the animals completed 90 days of life, considered then adult.

The mice were divided into four groups, which received different diets, as follows:

- GI group (n = 10) received the AIN-93M normocaloric diet;
- GII group (n = 10) was fed with the AIN-93M hyperlipidic diet;
- GIII group (n = 10) received the AIN-93M hyperlipidic diet + 0.5 mL of aqueous peanut extract daily;
- GIV group (n = 10) was fed with the AIN-93M hyperlipidic diet + 0.5 mL of aqueous peanut extract with 1% peanut skin.

The four groups received their experimental diets after 90 days of life (when the experiment started), with a daily consumption of these diets for a period of 12 weeks. From the time of weaning to 90 days, the animals received standard commercial food.

At the end of the experiment, the mice of all groups were submitted to intraperitoneal anesthesia with1 mL/kg of 2% xylazine solution (Rompun^®^), diluted in the proportion of 1:1 with ketamine (Fancotar^®^). One milliliter of blood was collected from each animal for laboratory tests. Then, the abdomen and thoracic cavity were fully opened for macroscopic analysis of the liver, heart and epididymal fat.

The liver, heart and epididymal fat of all animals were collected and weighed individually on a precision scale for comparison between groups. After weighing, the animals’ liver and heart were fixed in 10% buffered formaldehyde, remaining immersed for 24 h. Subsequently, the organs were included in paraffin and submitted to microtomy. The histological slides were then subjected to the staining technique using hematoxylin and eosin and analyzed under an optical microscope.

Each blood sample was centrifuged, and the plasma was used in the analysis of laboratory tests. In order to determine possible metabolic changes, serum concentrations of LDL, HDL, total cholesterol, triglycerides, C-reactive protein (PCR) and blood glucose were quantified. The biochemical parameters of the animals submitted to the different diets were compared with the group that received the AIN-93M normolipidic diet (GI).

To obtain the aqueous extract of peanuts with and without skin, the peanuts were peeled and immersed in clean water for 8 h. Then, it was separated into two parts: skinless peanut grain and peanut grain added with 1% peanut skin. The aqueous skinless peanut extract was prepared in a 1:8 ratio (peanut:water), in order to obtain the final concentration of 1.25 mg/mL. The aqueous skinned peanut extract was prepared in a similar way. However, in the extraction process, 1% of the peanut skin was added to the total volume.

The extraction was performed by the turbolization method, using a blender at a rotation of 6,000 rpmfor 3 min. The solvent used was distilled water, and, foreach 12.5 g of peanuts, 100 mL of it was used. Then, the extract was filtered through a simple filter^1^^,^ 9. The skinned and skinless peanut extract formulations were packaged in polypropylene packaging, sealed, and stored at -18±3°C.

The diet used in the research was purchased from a specialized laboratory and properly formulated to promote the increase of cholesterol and fat deposits in the animals’ organisms. Two types of feed were used: AIN-93M normolipid diet, formulated through the combination of purified ingredients in order to obtain a perfect nutritional balance for the animal; and the AIN-93M hyperlipidic diet, composed of the standard AIN-93M normocaloric diet added of 20% diet fat + 1% cholesterol + 0.5% cholic acid. Table 1 shows the nutritional values of the AIN-93M hyperlipidic diet administered to groups GII, GIII and GIV.

The analysis of the weight and biochemical parameters of the animals studied was conducted using the software Assistat^®^, version 7.4 beta, in which the data were subjected to analysis of variance (ANOVA) and the means, when necessary, compared by the Tukey’s test. Values with probability less than 0.05 (p<0.05) were considered significant. The construction of the graphs was performed through the GraphPad Prism^®^ software, version 5.

**Table 1:** Nutrition facts of the AIN-93M hiperlypidic diet.

Ingredients	p/kg
Corn starch	252.450 g
Casein	200 g
Dextrinized starch	132 g
Sucrose	100 g
Soy oil	40 g
Lard	160 g
Soluble fibers	50 g
L-cystine	3 g
Choline bitartrate	2.50 g
Butylated hydroxytoluene (BHT)	0.050 g
G mineral mix	35 g
Mix of vitamins	10 g
Cholesterol	10 g
Colic acid	5 g

p/Kg: amount of ingredients (in g) present in each kg of AIN-93Mhyperlipidic feed.

## Results

**Figure 1:**
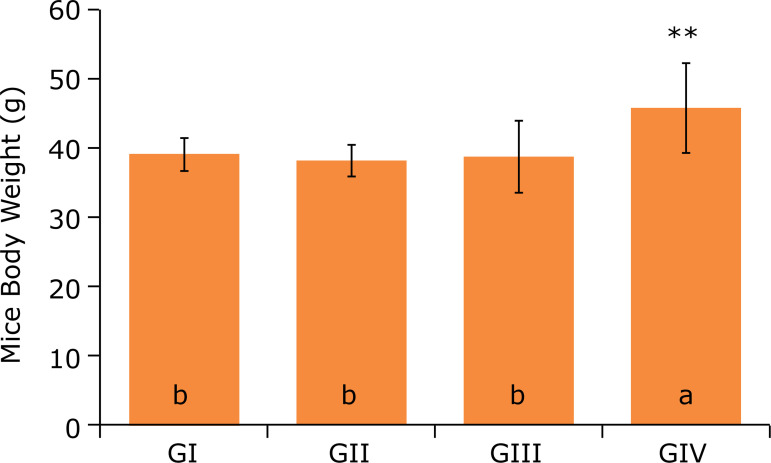
Mice body weight after 12 weeks of experiment^#^.

**Figure 2:**
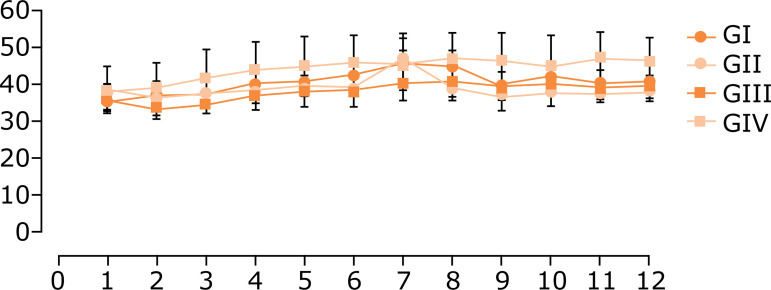
Evolution of mice body weight during the experiment.

GI: normocaloric diet group; GII: hyperlipidic diet group; GIII: hyperlipidic diet + 0.5 mL of peanut extract; GIV: hyperlipidic diet + 0.5 mL of peanut extract with 1% of peanut skin.

At the beginning of the experiment, the groups showed no statistical difference in weight between them, with the initial average of 37.1 g±2.61, 35.4 g±2.66, 35.8 g±2.32 and 38.5 g ± 4.21 (GI, GII, GIII and GIV, respectively). However, as shown in Fig. 1, at the end of 12 weeks, it was observed that the animals of the GIV group had a higher body weight when compared to the GI, GII and GIII groups. As presented in Fig. 2, the groups pointed out similar weight gain during the experiment.

**Statistically significant at the level of 1% probability (p<0.01); ^#^means followed by the same letter do not differ at 5% probability by the Tukey’s test; GI: normocaloric diet group; GII: hyperlipidic diet group; GIII: hyperlipidic diet + 0.5 mL of peanut extract; GIV: hyperlipidic diet + 0.5 mL of peanut extract with 1% of peanut skin.

**Figure 3:**
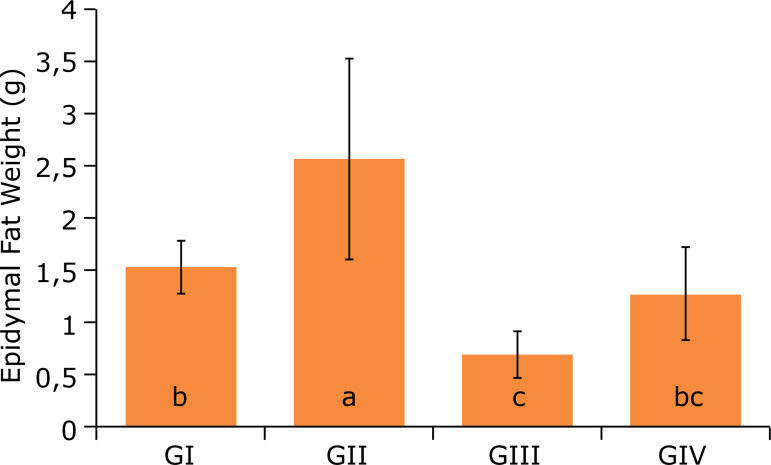
Epididymal fat weight after 12 weeks of experiment*.

**Figure 4:**
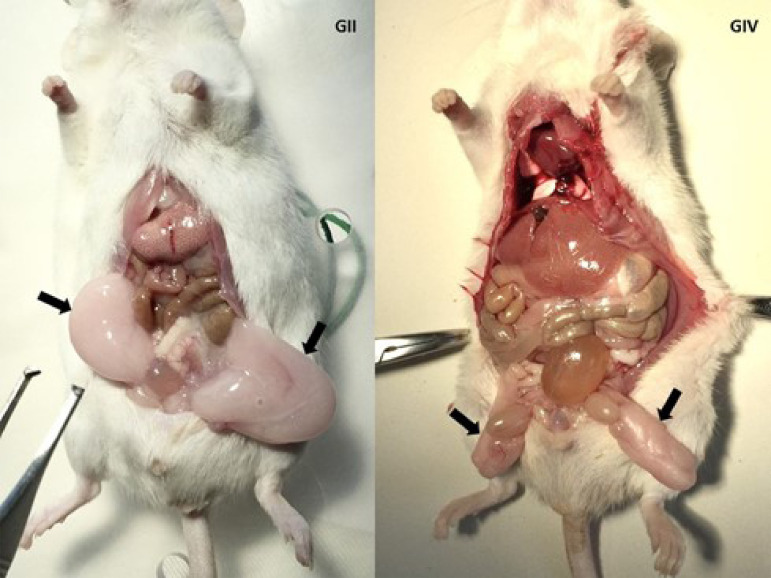
Macroscopic analysis of epididymal fat after 12 weeks of experiment.

In the analysis of the epididymal fat weight (Fig. 3), the groups GII and GIV did not present statistical difference between them, but a significant decrease in epididymal fat weight of GIII was observed. As seen in Fig. 4, after euthanasia of the animals, it was possible to verify that the group submitted to feeding with the AIN-93M hyperlipidic diet evolved with a considerable increase in the volume of the periepididymal lipid tissue when compared to the GIII.

The analysis showed p<0.01 between GII *vs*. GIII and p<0.05 between GII *vs*. GIV; GI: normocaloric diet group; GII: hyperlipidic diet group; GIII: hyperlipidic diet + 0.5 mL of peanut extract; GIV: hyperlipidic diet + 0.5 mL of peanut extract with 1% of peanut skin.

**Figure 5:**
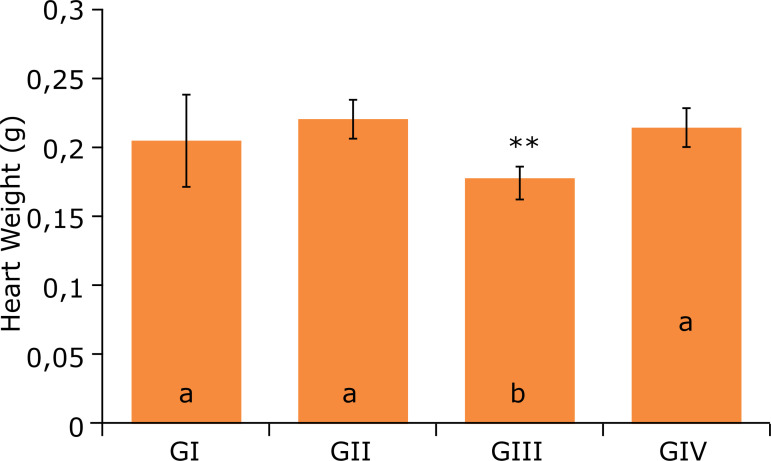
Heart weight after 12 weeks of experiment^#^.

**Figure 6:**
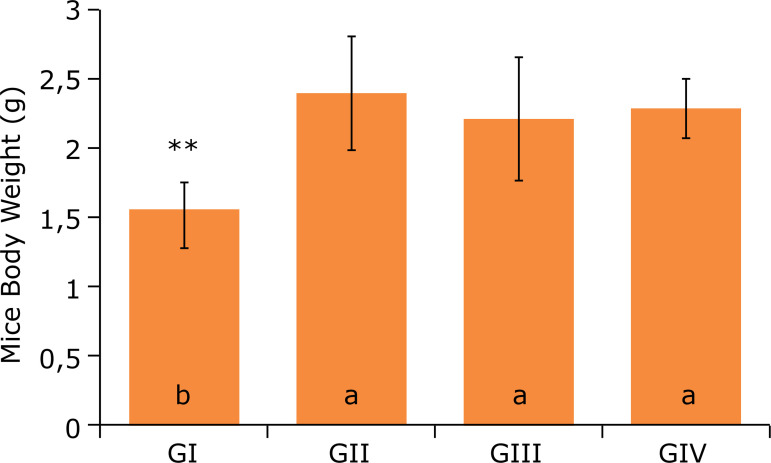
Liver weight after 12 weeks of experiment^#^.

Figures 5 and 6 show the analysis of the heart and liver weight of the four studied groups, respectively. The hyperlipidic diet changed the relative weight of the heart referring to GIII, whereas the weight of the liver among the groups did not undergo statistically significant changes, in accordance with previous studies^1^.

**The analysis showed p<0.01 between GI *vs*. GIII; ^#^means followed by the same letter did not differ at 5% probability by the Tukey’s test; GI: normocaloric diet group; GII: hyperlipidic diet group; GIII: hyperlipidic diet + 0.5 mL of peanut extract; GIV: hyperlipidic diet + 0.5 mL of peanut extract with 1% of peanut skin.

**The analysis showed p<0.01 between GI *vs*. GIII; ^#^means followed by the same letter did not differ at 5% probability by the Tukey’s test; GI: normocaloric diet group; GII: hyperlipidic diet group; GIII: hyperlipidic diet + 0.5 mL of peanut extract; GIV: hyperlipidic diet + 0.5 mL of peanut extract with 1% of peanut skin.

Table 2 shows the biochemical results obtained in the four experimental groups, evaluating the parameters of total cholesterol, glucose, HDL, very low-density lipoprotein (VLDL), LDL, triglycerides, and C-reactive protein (CRP). Increase in total cholesterol in GIII, increase in blood glucose in groups GII, GIII and GIV, decrease in serum LDL concentration in groups GI and GIV and increase in serum concentration of CRP in GII were seen. The results found for HDL, triglycerides and VLDL were not statistically significant between groups.

**Table 2:** Biochemical analysis after 12 weeks of experiment # .

	GI	GII	GIII	GIV	AM	CV%	p
Total cholesterol	106±9.20b	103±12.9b	126±12.7a	90±10.5b	106.5	10.86	**
Glucose	78±13.1c	225±26.7a	170±22.9b	194±32.41ab	167.2	14.86	**
HDL	96.6±9.20b	64.20±7.4b	56.30±4.2b	60.0±6.8b	69.2	19.7	ns
Triglycerides	47±11.66a	40±12.63a	50±22.12	45±18.32b	45.83	36.5	ns
VLDL	9.4±2.3a	8.1±2.5a	10.0±4.4a	9.0±3.6a	9.16	32.6	ns
LDL	10.4±2.8a	38.3±1.6b	56±2.2b	9.8±1.0a	28.6	11.54	**
CRP	0.57±0.10b	1.28±0.38a	0.96±0.21ab	0.93±0.23ab	0.93	44.6	**

HDL: high-density lipoprotein; VLDL: very low-density lipoprotein; LDL: low-density lipoprotein; CRP: C-reactive protein; AM: average mean; CV%: coefficient of variation (%);

** significant at the level of 1% probability (p<0.01); ns: not significant;

# means followed by the same letter did not differ at 5% probability by the Tukey’s test;

GI: normocaloric diet group; GII: hyperlipidic diet group; GIII: hyperlipidic diet + 0.5 mL of peanut extract; GIV: hyperlipidic diet + 0.5 mL of peanut extract with 1% of peanut skin.

**Figure 7:**
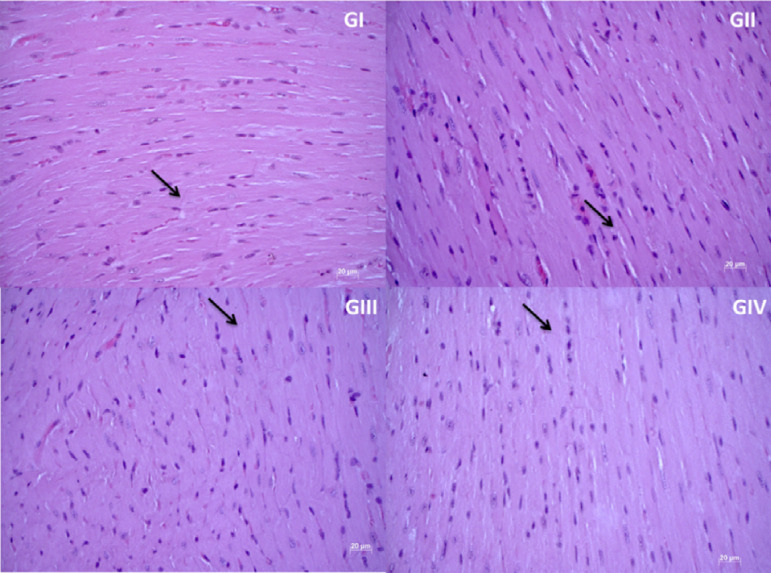
Photomicrograph of cardiac tissue, under hematoxylin and eosin staining and x40 objective.

**Figure 8:**
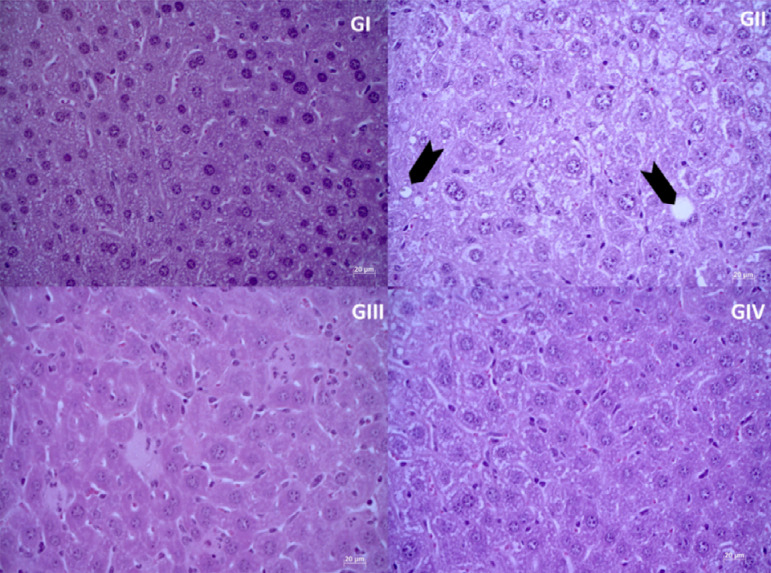
Photomicrograph of liver tissue, under hematoxylin and eosin staining and x40 objective.

Cardiac tissue was analyzed under light microscopy, observing the presence of well-preserved, healthy-looking cardiomyocytes, without significant cell damage or changes in all experimental groups (Fig. 7). In contrast, after the histopathological assessment of the GII animals, the presence of vacuolar fat deposits with a degenerative appearance similar to mild non-alcoholic fatty liver disease was found (Fig. 8 GII). The animals in the other experimental groups did not show liver changes in histological analysis. The livers of animals in groups GI, GIII and GIV were homogeneous in appearance, integrity of the liver lobes and the portal space, with well-defined liver veins and sinusoidal cords present, which were intact and converging into the central-lobular vein (Fig. 8GI, GIII, GIV). There were no signs of liver inflammation in any of the four groups.

*Arrows:* intact intercalated discs; GI: normocaloric diet group; GII: hyperlipidic diet group; GIII: hyperlipidic diet + 0.5 mL of peanut extract; GIV: hyperlipidic diet + 0.5 mL of peanut extract with 1% of peanut skin.

*Large arrows:* vacuolar degeneration of hepatocytes, indicating lipid deposit; GI: normocaloric diet group; GII: hyperlipidic diet group; GIII: hyperlipidic diet + 0.5 mL of peanut extract; GIV: hyperlipidic diet + 0.5 mL of peanut extract with 1% of peanut skin.

## Discussion

The mice body weight measures found in this work disagree with Wang et al., who, after treating two groups of mice with a high-fat diet, showed that the body weight of the mice in the control group was 62.98% higher than the group with chitosan intervention^10^. However, these authors used only the morphometric parameter of body weight and concluded that the high-calorie diet was effective in promoting obesity in this animal model. It was expected that animals in the GII group had higher weight values than those in the GI group. However, it was observed that the animals in the group that received a high-calorie diet had the final body weightof 38.04 ± 2.38 g, and the animals in the group with a normolipidic diet, 39.01 ± 2.30 g.

The group that received AIN-93M hyperlipidic diet + 0.5 mL of aqueous peanut extract added with 1% of peanut skin obtained a higher average weight(45.71 ± 5.2) with a difference of at least 14.67% when compared to GI. This increase may be related to muscle mass gain, since the aqueous extract of peanuts has a high-protein index when compared to other vegetable drinks.

Epididymal fat is a relevant parameter to identify the percentage of fat in males^11^^,^ 12. A significant volume of epididymal fat was found in GII group, which is a result of the hypercaloric consumption of the administered diet.

In body homeostasis, lipid metabolism maintains a balance between synthesis and degradation. When the synthesis is greater than the degradation, there is the development of dyslipidemia, which can progress with peripheral arterial disease and even acute coronary syndrome in the most severe cases^11^^,^ 13. In its measurement, in GIII total cholesterol (126 ± 12.7) was statistically higher when compared to GI, GII and GIV.

According to the biochemical analysis, it was possible to observe that the peanut did not interfere in the levels of total cholesterol, HDL, and triglycerides. However, it reduced in GI and GIV the LDL levels, corroborating with the study by Ma *et al*.^4^, which concluded that peanuts and peanut butter are cholesterol-free and can help to reduce serum LDL levels and the risk of cardiovascular diseases.

The CRP is produced in the liver, and its blood concentration rises when an inflammatory process takes place. The high serum concentrations of CRP in adult individuals with metabolic syndrome are a strong relationship between the accumulation of visceral fat and increased LDL levels. The accumulation of fatty acids in the blood tissue suggests that there is a tendency to oxidative damage and destabilization of homeostasis in the metabolism, providing an inflammatory process^5^^,^ 14.

Regarding CRP, increase in the GII group was observed, which can be justified by the greater availability of fat offered to the animals. The non-change in the CRP concentrations of GIII and GIV, which received the aqueous peanut extract, can be explained by the number of polyphenols present in the extracts, protecting the lipidic metabolism of mice^5^.

The reduction of LDL levels in the GI and GIV groups is compatible with the results of the CRP, suggesting reduction in cardiovascular risk, since high concentrations of this lipoprotein are related to a greater propensity to cardiovascular diseases. The reduction in LDL levels in animals treated with peanut extract add of 1% peanut skin can be attributed to the chemical composition of the peanut skin, which is rich in dietary fiber and compounds with antioxidant action^14^.

It was observed that the groups that received a high-fat diet add of aqueous peanut extract with and without skin showed no significant difference between them in blood glucose values. However, the results are different when compared to GI, suggesting that the increase in blood glucose in animals is related to the high-serum cholesterol concentrations, without the influence of aqueous peanut extract^15^^,^ 16.

As the liver is the organ with the greatest metabolic power, its analysis is a great way to assess the effect of drugs, toxins, and other physiological responses. Because it has the function of converting and storing biomolecules, the liver tissue has a more sensitive response to the effects of the high-fat diet, due to gluconeogenesis. It is known that peanuts have compounds such as resveratrol, phenolic acid, flavonoids and phytosterols, which inhibit the absorption of dietary cholesterol. In addition, peanuts are a source of Co-enzyme Q10, contain all 20 amino acids, and are a source of antioxidants that act to protect against oxidative stress^17^.

The vacuolar damage identified in the hepatocytes of animals in the experimental group that received a high-fat diet may justify the increased plasma levels of glucose and LDL, both processed in this tissue. However, the group that received the addition of the aqueous peanut extract associated with the high-fat diet showed improvements in histopathological evaluation, even though plasma changes were still perceived, which suggests a beneficial effect of peanuts on tissues during continued consumption.

In the group that received the peanut extract add of 1% peanut skin, even with consumption of a high-fat diet, plasma characteristics improved more when compared to other groups that also had a high-fat diet. Beneficial effects of peanut oils from different forms of extraction have improved blood lipid levels and other biochemical parameters in rats^10^.

## Conclusions

The aqueous peanut extract improved the plasma concentrations of LDL of the animals that received a high-fat diet and avoided morphological damage of the liver tissue. Such benefits were intensified by associating peanut skin with aqueous extract in animals that received a high-fat diet. These results may have a broader implication in humans for their use in the prevention of dyslipidemia and obesity-related disorder, with a significant therapeutic potential for using peanut skin as an added-value ingredient in peanut-based products.
